# Gold nanoparticles delivered miR-375 for treatment of hepatocellular carcinoma

**DOI:** 10.18632/oncotarget.13431

**Published:** 2016-11-17

**Authors:** Hui-Ying Xue, Yong Liu, Jia-Zhi Liao, Ju-Sheng Lin, Bin Li, Wei-Gang Yuan, Robert J. Lee, Lei Li, Chuan-Rui Xu, Xing-Xing He

**Affiliations:** ^1^ Institute of Liver Diseases, Tongji Hospital, Tongji Medical College, Huazhong University of Science and Technology, Wuhan 430030, China; ^2^ School of Pharmacy, Tongji Medical College, Huazhong University of Science and Technology, Wuhan 430030, China; ^3^ Division of Pharmaceutics and Pharmaceutical Chemistry, College of Pharmacy, The Ohio State University, Columbus, Ohio, USA

**Keywords:** microRNA, liver cancer, nanomedicine, therapy, AEG-1

## Abstract

MiR-375 is a tumor suppressor miRNA that is downregulated in hepatocellular carcinoma (HCC). However, due to the lack of effective delivery strategies, miR-375 replacement as a therapy for HCC has not been investigated. In the present study, we have developed a straightforward strategy to deliver miR-375 into HCC cells by assembling miR-375 mimics on the surface of AuNPs and forming AuNP-miR-375 nanoparticles. AuNP-miR-375 exhibits high cellular uptake and preserves miR-375′s activities to suppress cellular proliferation, migration/invasion, and colony formation, and to induce apoptosis in HCC cells. Furthermore, AuNP-delivered miR-375 efficiently downregulated its target genes through RNA interference. In primary and xenograft tumor mouse models, AuNP-miR-375 showed high tumor uptake, therapeutic efficacy, and no apparent toxicity to the host mice. In conclusion, our findings indicate that AuNPs is a reliable strategy to deliver miR-375 into HCC cells and tissue, and that AuNP-miR-375 has the potential in the clinic for treatment of unresectable HCC.

## INTRODUCTION

Hepatocellular carcinoma (HCC) is the most common form of liver cancer and one of leading causes of cancer related deaths worldwide. Given that most HCCs are diagnosed at advanced stages, treatment options for HCC are limited [1]. Therefore, there is an unmet clinical need for development of novel effective treatments for HCC.

MicroRNAs (miRNAs) are small non-coding RNAs that function as master regulators of gene expression. Dysregulated miRNAs play critical roles in the tumor initiation and progression in many types of cancers. Among them, down-regulation of miR-375 has been reported in various tumors, including HCC, gastric cancer, esophageal cancer, pancreatic cancer and so on [2–6]. Previously, we found that miR-375 is significantly down-regulated in HCC cell lines and tissues, and demonstrated that miR-375 suppresses malignant traits of HCC by targeting AEG-1 and ATG7 [2, 7]. Furthermore, we found that the hypermethylation of CpG islands in miR-375 promoter region may have led to the down-regulation of miR-375 in HCC [8]. Additionally, Liu et al. found that miR-375 was notably down-regulated in HCC and increasing miR-375 expression decreased HCC cell invasion and proliferation by targeting oncogene YAP1 [9]. Furthermore, miR-375 was recently found to strongly inhibit Akt/Ras induced hepatocarcinogenesis in a primary mouse model of liver cancer [10]. Collectively, these studies suggest that miR-375 is an attractive therapeutic target for HCC.

Recently, there has been an unprecedented expansion in the field of nanomedicine with the development of new nanoparticles for the treatment of cancers [11]. Gold nanoparticles (AuNPs) are particularly promising because they are biocompatible, easy to be surface functionalized, and suitable for carrying small molecule as well as macromolecule drugs such as DNA or RNA [11, 12]. Polyvalent oligonucleotide-functionalized AuNPs have several properties that make them ideal for biomedical applications [13]: i) AuNPs have the capability to enter cultured cells or animal tissues without the aid of lipid or polymer-based co-carriers [14]; ii) oligonucleotides on their surfaces are resistant to nuclease degradation and, therefore, more stable than the same sequences free in solution [15]; and iii) innate immune response elicited by these conjugates is significantly lower than the same DNA delivered using commercial lipid-based carriers [16, 17]. AuNPs have been evaluated as drug carriers and as sensitizers in photothermal therapy. However, AuNPs has not been explored for delivery of miRNAs for HCC treatment.

**Figure 1:**
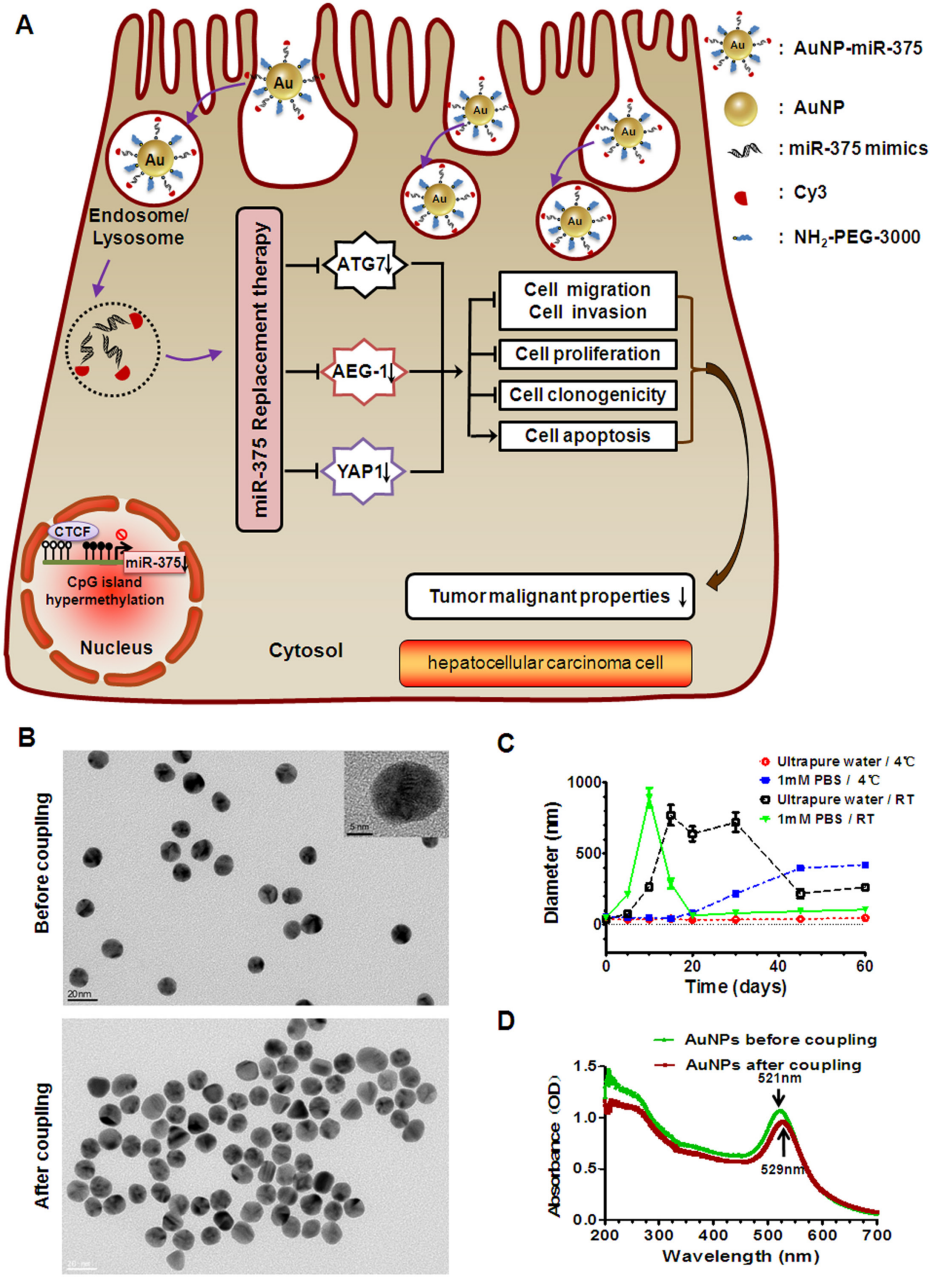
Proposed mechanism for cellular uptake of AuNP-miR-375 nanoparticles and characteristics of the NPs **A**. Schematic illustration of gold nanoparticles delivered miR-375 for replacement therapy in hepatocellular carcinoma (HCC). After being taken up by hepatoma cells, AuNP-miR-375 nanoparticles escape from endosome/lysosome and mature miR-375 is released to the cytoplasm. Released miR-375 acts on its target genes like AEG-1, YAP1, and ATG7, and suppresses tumor malignant phenotypes of HCC cells. **B**. The shape of AuNP and AuNP-miR-375 nanoparticles imaged by a transmission electron microscope (TEM). **C**. The stability of AuNPs in ultrapure water and phosphate buffered saline (PBS) under 4°C or room temperature (RT). The hydrodynamic diameters of AuNPs were measured 8 times during more than two months by dynamic light scattering (DLS). **D**. Absorbance specturm of AuNP and AuNP-miR-375 nanoparticles obtained using a UV-Vis spectrophotometer.

Herein, we designed and prepared an AuNP system to deliver miR-375 for miRNA replacement therapy in HCC (see schematic illustration in Figure 1A). A double stranded miR-375 mimic was covalently linked to AuNPs to form AuNP-miR-375. The nanoparticles exhibited high cellular uptake efficiency, successfully preserved miR-375′s biological function. In primary and xenograft tumor mouse models, AuNP-miR-375 was shown to be highly effective as miR-375 replacement therapy without apparent toxicity to host mice.

## RESULTS

### Preparation and characterization of AuNPs and AuNP-miR-375

AuNPs and their miR-375 conjugates AuNP-miR-375 were prepared as described in the methods section. A miR-375 mimic, labeled with Cy3 at the 3’ end of the antisense strand, was linked to AuNPs covalently through a gold-sulfur bond, and can be easily tracked by fluorescence imaging. An animated PEG layer on the particle surface was used to stabilize AuNP-miR-375. AuNPs are spherical in shape and have a mean diameter of approximately 13 nm as measured by TEM (Figure 1B) and a mean hydrodynamic diameter of 36 ± 3 nm as measured by DLS (Table 1). Their wavelength of absorption was centered at 521 nm according to the UV-Vis spectrum (Figure 1D). We compared the stability of AuNPs in ultrapure water and in PBS under 4°C or room temperature (RT), and found that AuNPs stored in ultrapure water at 4°C were relatively more stable, with little change in hydrodynamic diameter in more than two months (Figure 1C). After conjugation to miR-375 and PEG, AuNP-miR-375 showed no significant change in morphology based on TEM images (Figure 1B), but had increased sizes, with a mean hydrodynamic diameter of 53 ± 8 nm by DLS (Table 1). In addition, AuNP-miR-375 showed a maximum absorption peak at 529 nm, indicating a red shift caused by miR-375 conjugation (Figure 1D). Zeta potential of AuNPs was -55 ± 0.7 mv, and it increased to -34 ± 1.8 mv in AuNP-miR-375 (Table 1). The increase in Zeta potential was due to charge density reduction since RNA grafted on the Au surface and acted as a non-associated surface passivation layer. The polydispersity of AuNPs was 0.197 ± 0.034 and it increased slightly to 0.321 ± 0.191 in AuNP-miR-375, suggesting that both AuNPs and AuNP-miR-375 had narrow size distribution. Taken together, these results indicated that AuNP-miR-375 was successfully synthesized and had the expected properties.

**Table 1:** Characteristics of AuNPs determined by dynamic light scattering (DLS)

Groups	Particles size (nm)	Polydispersity	Zeta potential (mV)
AuNPs	36 ± 3	0.197 ± 0.034	- 55 ± 0.7
AuNP-miR-375	53 ± 8	0.321 ± 0.191	- 34 ± 1.8

### Cellular uptake of AuNP-miR-375 and release of miR-375 in hepatoma cells

**Figure 2:**
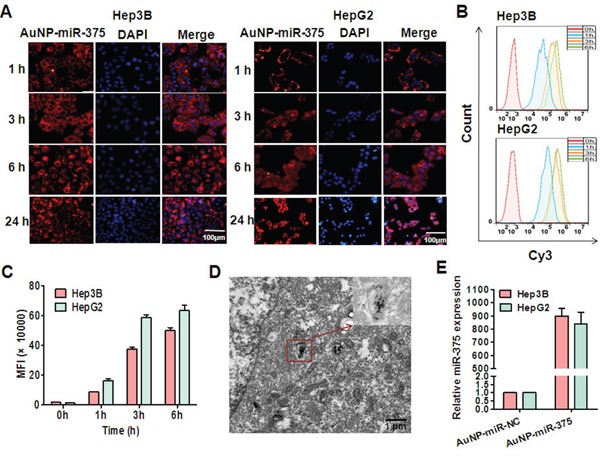
Cellular uptake of AuNP-miR-375 in hepatoma cells and release of miR-375 **A**. Cellular uptake of AuNP-miR-375 in HepG2 and Hep3B cells visualized by fluorescence microscope. Hepatoma cells were incubated with Cy3-labeled AuNP-miR-375 (50 nM miR-375) for different time and then stained with DAPI and observed under a fluorescence microscope. **B**. Cellular uptake of AuNP-miR-375 in HepG2 and Hep3B cells determined by flow cytometry analysis. HepG2 and Hep3B cells were treated with AuNP-miR-375 (50 nM miR-375). **C**. Mean fluorescence intensity was determined by flow cytometry analysis. **D**. MiR-375 localization in endosome/lysosomes in HepG2 cells. HepG2 cells were incubated with AuNP-miR-375 in 10 cm culture dishes for 6 h and then observed under a transmission electron microscope (TEM). **E**. The expression levels of mature miR-375 in hepatoma cells were detected by TaqMan qRT-PCR.

To study whether AuNPs can deliver miRNA into liver cancer cells efficiently, we determined the cellular uptake of Cy3-labeled AuNP-miR-375 by fluorescence microscopy and by flow cytometry analysis. After 1 h incubation, Hep3B and HepG2 cells treated with AuNP-miR-375 showed significant red fluorescence in almost all cells, indicating efficient and rapid uptake of AuNP-miR-375 by the cells (Figure 2A). Stronger fluorescence was observed in the cells at 3, 6 and 24 h post treatment (Figure 2A). To quantify the fraction of cells taking up AuNP-miR-375, we used flow cytometry analysis to detect Cy3-positive cells. After 1 h treatment, more than 95% Hep3B cells were Cy3-positive, indicating cellular uptake of AuNP-miR-375 (Figure 2B). Increased fluorescence was detected in Hep3B cells at 3 h, and fluorescence were increased only slightly from 3 to 6 h, suggesting that the uptake of AuNP-miR-375 was saturated (Figure 2C). Examination under TEM showed that AuNP-miR-375 particles were localized in endosomes or lysosomes (Figure 2D). Furthermore, we used TaqMan qRT-PCR to detect release of mature miR-375 in hepatoma cells and found that expression of miR-375 was increased nearly 900 times in Hep3B and HepG2 cells treated with AuNP-miR-375 (Figure 2E). These results suggested efficient cellular uptake of AuNP-miR-375 and release of intact mature miR-375 in HCC cells.

### AuNP-miR-375 suppresses tumor cell phenotypes in vitro

**Figure 3:**
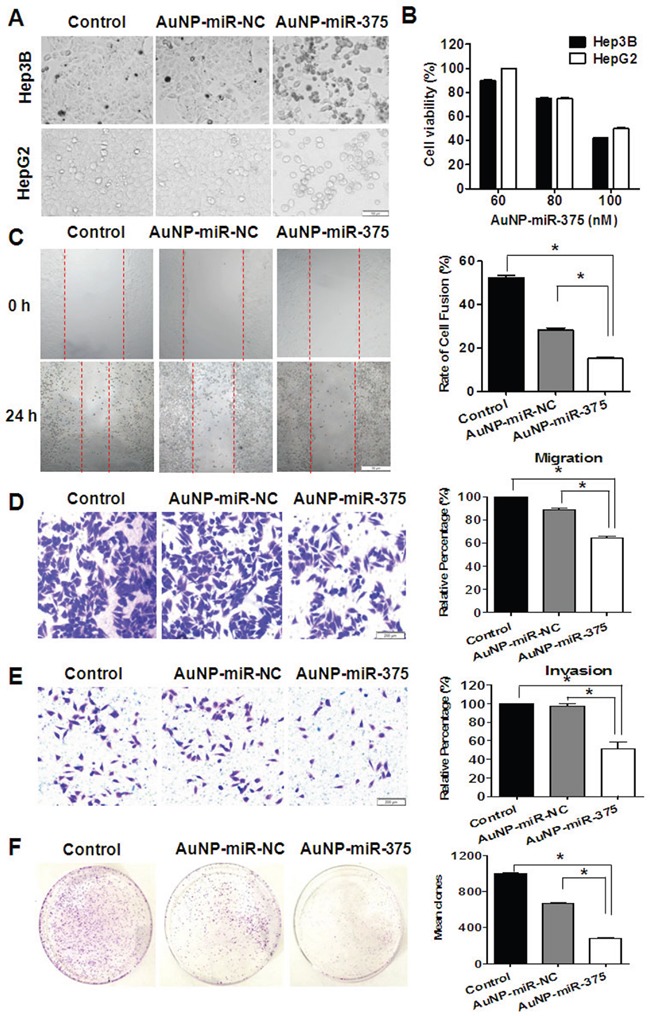
Anti-tumor activities of AuNP-miR-375 in hepatoma cells **A**. Morphology and cell viabilities were measured in HepG2 and Hep3B cells treated with AuNP-miR-375 (100 nM miR-375) or the controls for 48 h. The pictures were taken under an inverted light microscope (× 100). **B**. Viabilities of HepG2 and Hep3B cells treated with AuNP-miR-NC or AuNP-miR-375 for 48 h at different concentration of miR-375 as indicated. **C**. Wound-healing of Hep3B cells treated with AuNP-miR-NC or AuNP-miR-375 for 24 h. Quantitative results were calculated as: percent closure (%) = length of cell migration (mm) / width of wounds (mm) × 100%. Percent closure of control group was standardized as 100%. **D**. Transwell assay of Hep3B cells treated with AuNP-miR-NC or AuNP-miR-375. **E**. Matrigel invasion assay of Hep3B cells treated with AuNP-miR-NC or AuNP-miR-375. **F**. Colony formation assay of Hep3B cells. Hep3B cells were seeded in culture dishes for 2.5 × 10^3^ per well and allowed to grow for 15 days. All data are shown as mean ± SEM of 3 independent experiments. *: P < 0.05.

**Figure 4:**
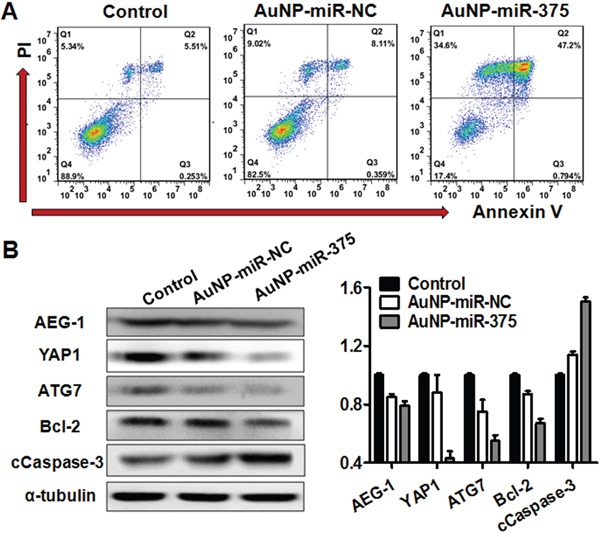
AuNP-miR-375 induced cell apoptosis in Hep3B cells and acted on miR-375′s downstream genes **A**. Apoptosis determined by flow cytometry analysis in Hep3B cells treated with AuNP-miR-375. Hep3B cells were treated with AuNP-miR-NC or AuNP-miR-375 (100 nM miR-375) for 48 h. Quadrants from lower left to upper left (counter clockwise) represent healthy, early apoptotic, late apoptotic, and necrotic cells, respectively. The percentages of cells in each quadrant were shown on the graphs. **B**. AEG-1, YAP1, ATG7, Bcl-2, and cleaved Caspase 3 proteins were measured by western blot analyses 48h after treatment. α-tubulin was used as a loading control. For quantification of the western blotting bands, pictures were analyzed software Image J (version 2.0) and data are expressed as mean ± SEM of 3 scanning.

Cell proliferation, migration/invasion, colony formation, and apoptosis were investigated to determine the effects of AuNP-miR-375 in vitro. Firstly, we found that AuNP-miR-375 induced significant cell growth inhibition and cell death in HepG2 and Hep3B cells based on microscopic cell counting (Figure 3A). Then, cell proliferation with various concentration of AuNP-miR-375 was measured using a CCK-8 based growth detection kit. As shown in Figure 3B, cell growth inhibition in HepG2 or Hep3B cells was increased along with escalation of AuNP-miR-375 dose. To determine the effect of AuNP-miR-375 on the motility of HCC cells, we performed wound-healing and migration/invasion assays. The results from the wound-healing assay showed that the wounds were closed about 50% in untreated cells, 28% in AuNP-miR-NC treated cells, and about 15% in Au-NP-miR-375 treated cells, indicating that AuNP-miR-375 decreased the motility of HCC cells (Figure 3C). Similarly, we found in a transwell assay that AuNP-miR-NC and AuNP-miR-375 treatment resulted in significant migration inhibition, leading to 15% and 40% reduction in migration compared to control, respectively (Figure 3D). Transwell assay showed that AuNP-miR-375 could block the invasion of Hep3B cells (Figure 3E). Furthermore, colony formation assay indicated that tumorigenic capability of HCC cells was significantly impaired by AuNP-miR-375 treatment (Figure 3F). Finally, flow cytometry analysis of cell apoptosis showed that AuNP-miR-375 induced apoptosis in more than 47% of Hep3B cells, whereas AuNP-miR-NC treated group showed only a weak induction of apoptosis in Hep3B cells (Figure 4A). These results collectively confirmed that miR-375 delivered by Au-NPs could suppress tumor cell proliferation and migration in vitro.

### AuNPs delivered miR-375 can act on its downstream gene targets

To investigate whether the AuNPs interfere with the working of miR-375, we determined the expression of miR-375′s downstream gene targets in Hep3B cells treated with AuNP-miR-375. Previous studies have shown that AEG-1, YAP1 and ATG7 are three major targets of miR-375 in HCC [2, 7, 9]. Additionally, AEG-1 has been reported to regulate Bcl-2 and Caspase-3 to prevent cellular apoptosis [18]. Thus, we detected the expression of those genes using western blotting and found that expression of AEG-1, YAP1 and ATG7 were significantly reduced in Hep3B cells after AuNP-miR-375 treatment (Figure 4B). Correspondingly, Bcl-2 protein expression was remarkably decreased and cleaved Casepase-3 expression was significantly increased (Figure 4B). This result indicates that AuNPs in the miRNA conjugates did not affect the biological function of miR-375.

### Tissue distribution and toxicity of AuNP-miR-375

**Figure 5:**
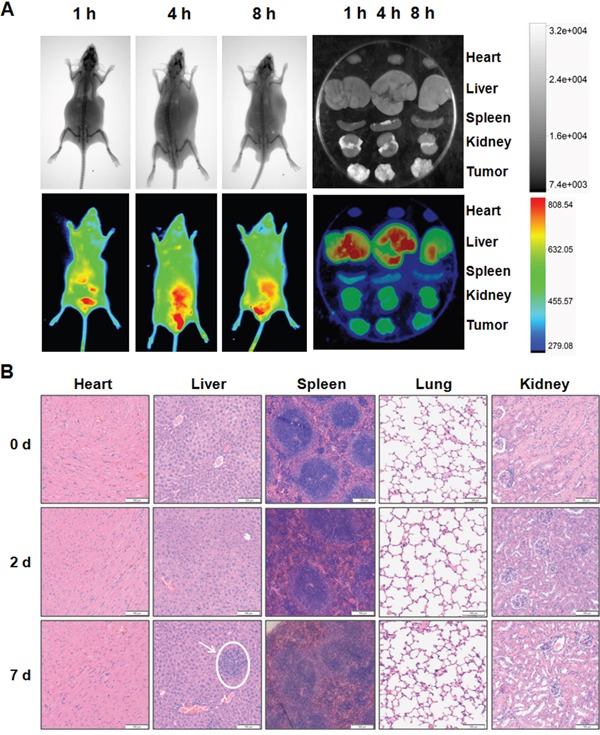
Tissue distribution and toxicity of AuNP-miR-375 **A**. Distribution of miR-375 delivered by AuNP-miR-375 in xenograft mice. Tumor bearing nude mice were injected via tail vein with a single dose AuNP-miR-375 (4 nmol/kg miR-375) when tumors grew to ∼500 mm^3^. X-ray (upper row) and bioluminescence were performed on mice injected with AuNP-miR-375 after anesthesia. **B**. Histological analysis of hearts, livers, spleens, lungs and kidney from mice treated with AuNP-miR-375. FVB/N mice were given a single injection of AuNP-miR-375 (8 nmol/kg miR-375) via tail vein. Mice were sacrificed at 0, 2 and 7 days, respectively, and tissues were collected for histological analysis.

To explore whether miR-375 can be efficiently and specifically delivered into tumor tissues by AuNP-miR-375, we examined the distribution of Cy3-labeled miR-375 in tumor bearing BALB/c nude mice following i.v. administration. The mice were given a single injection of AuNP-miR-375 via tail vein and then examined using a small animal in vivo imaging system. Observations revealed that miR-375 appeared in tumor tissues at 1 h post injection (red circle) and the peak time was around 4 h after injections (Figure 5A). In addition, miR-375 showed high accumulation in the liver, which potentially favors its efficacy in HCC treatment (Figure 5A). Imaging of isolated organs showed the distribution of miR-375 in tumor tissue more clearly than in vivo imaging (Figure 5A). Interestingly, miR-375 showed significant clearance from the liver at 8 h post injection, but a high accumulation in the tumor tissue. These results demonstrate that AuNPs can efficiently and specifically deliver miR-375 into tumor tissues and maintain elevated miRNA concentration in these tissues.

Safety is a critical issue in drug development, especially for drugs administrated through systemic administration. To evaluate the safety of AuNP-miR-375, we injected mice with AuNP-miR-375 intravenously via tail vein and then observed for 7 days. No death or adverse effects were observed in 7 days following drug administration. No apparent organ injury or bleeding was found through dissection of those mice. Histological examination of the mouse heart, liver, spleen, lung and kidney revealed that minor acute inflammation appeared in the liver but not in other organs (Figure 5B). In addition, AuNP-miR-375 administration resulted in a minor reduction in white pulp in spleens, but did not lead to severe injury. Overall, these results indicate that miR-375 can be efficiently and specifically delivered into tumor tissues by AuNP-miR-375, and that AuNP-miR-375 has negligible side effects in the treatment of liver cancer.

### AuNP-miR-375 suppressed tumor growth in primary and xenograft tumor mouse models

**Figure 6:**
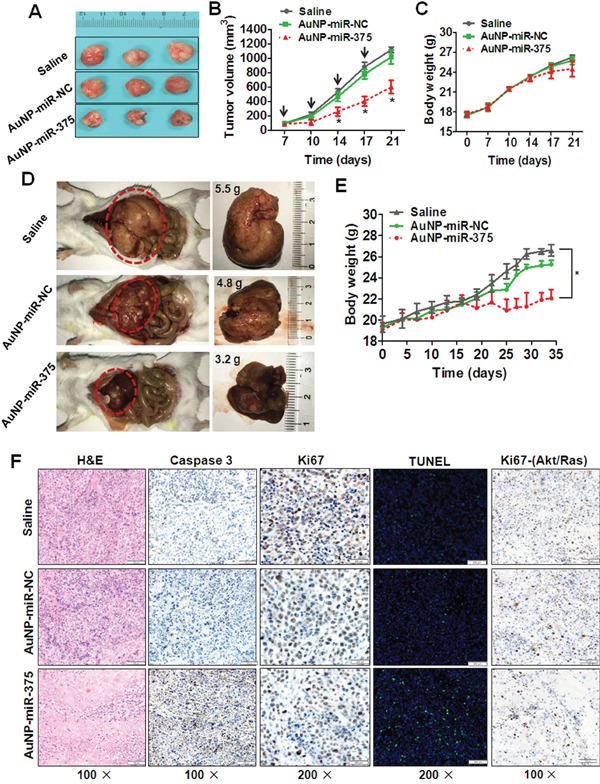
Anti-tumor effects of AuNP-miR-375 in xenograft and primary HCC mouse models **A**. Effects of AuNP-miR-375 on the growth of pre-established HepG2 xenografts at a gross morphology level. Subcutaneous tumors were seeded by inoculation of 5 × 10^6^ HepG2 cells in the front armpit of the mice. At the tumor volume of 100 mm^3^, AuNP-miR-375 was injected via intra-tumor injection as described in the method section. At day 21 after HepG2 cells inoculation, mice were sacrificed and tumors were collected and photographed. **B**. Tumor growth curve of mice with pre-established HepG2 xenografts treated with AuNP-miR-NC or AuNP-miR-375. **C**. Body weights of mice with xenografts treated with AuNP-miR-NC or AuNP-miR-375. **D**. Tumor morphology and sizes in mice with primary HCC treated with AuNP-miR-NC or AuNP-miR-375. Akt and Ras plasmids were hydrodynamicly injected into 6 weeks old FVB/N mice via tail vein, and tumors were generally formed in the livers 2 weeks post plasmid injection. Then those mice were given injections of AuNP-miR-NC or AuNP-miR-375 (4 nmol/kg miR-375), once every other day for total 3 weeks. At the end of experiment, the mice were sacrificed and the livers were photographed. **E**. Body weight of mice with primary liver tumor treated with AuNP-miR-NC or AuNP-miR-375. **F**. Hematoxylin and eosin staining, immunohistochemical analysis, and TUNEL Staining of tumor tissues in xenograft mice and primary HCC mice. Xenograft tumor tissues were excised, fixed and then examined by hematoxylin and eosin staining, immunohistochemical analysis of Caspase-3 and Ki67, and TUNEL Staining. Primary tumor tissues from Akt/Ras induced HCC in mice were excised, fixed and then immunostained with antibodies against Ki67. *: P < 0.05.

To determine the anti-tumor effect of AuNP-miR-375 in HepG2 xenograft tumor mouse model, AuNP-miR-375 was administrated by intratumoral injection upon the subcutaneous tumor reaching ∼ 100 mm^3^. AuNP-miR-375 showed marked suppression of growth of xenograft tumors, as shown by gross morphology (Figure 6A) and growth curves (Figure 6B). We also examined body weights of mice after drug injections. The results showed no significant difference between AuNP-miR-375 and AuNP-miR-NC treatment groups (Figure 6C).

We then studied the therapeutic activity of AuNP-miR-375 in primary HCC tumors. To mimic HCC pathology in human patients, an Akt/Ras induced primary HCC mouse model was generated by hydrodynamic injection of plasmids carrying these two genes into mice as described previously [19]. Akt promotes lipogenesis and Ras drives mitosis in hepatocytes. Synergetic effects of these two genes lead to tumor formation in livers [19]. Two weeks after Akt/Ras injection, tumors formed in mice and then AuNP-miR-375 was administrated by tail vein injection. After administration of 10 doses, liver weights of mice treated with AuNP-miR-375 were much smaller than those of the controls, with average weights of 3.2, 4.8 and 5.5 g, respectively (Figure 6D). Importantly, livers in saline or AuNP-miR-NC treated mice were more inhomogeneous in color, were paler, and had more nodular lesions than those in AuNP-miR-375 treated mice. Correspondingly, AuNP-miR-375 treated mice have more normal liver tissues compared with AuNP-miR-NC treated mice (Figure 6D). Accordingly, body weights of mice treated with AuNP-miR-375 was also much lower than those of mice treated with AuNP-miR-NC, suggesting a reduction of tumor burden in mice (Figure 6E).

At the end of the experiment, resected tumor tissues from those treated xenograft tumors and primary tumors were collected for histological examination. In xenograft tumor tissues, the expression of Ki67 in AuNP-miR-375 treated xenograft tumors was much lower than that in AuNP-miR-NC or saline treated xenograft tumors (Figure 6F). Correspondingly, AuNP-miR-375 treatment induced higher level of expression of cleaved Caspase-3 (Figure 6F). Moreover, TUNEL assay showed more apoptotic cells present in AuNP-miR-375 treated xenograft tumors than in AuNP-miR-NC treated xenograft tumors (Figure 6F). Similarly, Ki67 expression was also down-regulated in AuNP-miR-375 treated primary HCC tumor tissues (Figure 6F). These results indicate that cell proliferation was inhibited and cell apoptosis was induced in tumors treated with AuNP-miR-375.

Taken together, these results from xenograft and primary tumor mouse models clearly demonstrated potent therapeutic effects of AuNP-miR-375 in vivo, suggesting that AuNP is an effective carrier for miRNA delivery and that AuNP-miR-375 is a potential agent for HCC treatment.

## DISCUSSION

Growing evidences have demonstrated that miR-375 functions as an important tumor suppressor in HCC and represents a promising candidate for miRNA replacement therapy due to its capacity to inhibit tumor cell growth in vitro and in vivo [2, 6, 7, 9]. However, the lack of an efficient delivery system has been a major obstacle impeding the therapeutic application of miR-375 in HCC. In this study, we developed an AuNP-miRNA delivery system to deliver miR-375 into HCC cells and tissues and proved the therapeutic effect of miR-375 in HCC.

Our study indicated that miR-375 delivered by nanoparticle delivery system could enter HCC cell or tissues and function as a tumor suppressor. Uptake experiments revealed that AuNP-miR-375 could efficiently deliver miR-375 into HCC cell and tissues. Delivered miR-375 significantly downregulated its downstream target genes, suggesting that AuNPs delivered miR-375 has same biological function as endogenous miR-375. Morphological analysis revealed that delivered miR-375 can function as tumor suppressor in HCC cells, indicating that artificial replacement of miR-375 could restore the regulating networks of miR-375. These results are in agreement with our previous research, in which overexpression of miR-375 in hepatoma cells by transfection of miR-375 precursor with Lipofectamine™ 2000 inhibited tumor growth [2]. Importantly, our results from primary and xenograft tumor mouse models also indicate that AuNP-miR-375 can overcome physiological obstacles and deliver miR-375 into HCC tissues in mice. Highly distributed AuNP-miR-375 in HCC tissues showed increased anti-tumor activity compared with AuNP-miR-NC (Figure 5).

Our study also suggested that AuNPs can serve as an attractive platform for nucleic acid delivery in cancer treatment. First, our results indicated that AuNPs are biocompatible and non-toxic. Delivery of AuNPs to mice did not induce any severe injury in mice. Second, AuNPs are easy modified to deliver nucleic acid. MiRNA or siRNA was easily to be conjugated to AuNPs through thiol bond. High uptake efficiency and release of intact miR-375 suggests that AuNPs are indeed apt to be taken up by HCC cells and protect miRNA from degradation. Finally, AuNP itself possess anti-tumor effect and may improve the efficacy of delivered anti-tumor agents. In our study, our results clearly showed that AuNP-miR-NC showed miminal anti-tumor effect in comparison with negative control. Hence, AuNPs represent a promising and potent delivery system for nucleic acid.

In conclusion, our study illustrated the reliability of AuNPs to deliver miR-375 into HCC cells and the therapeutic effects of AuNP-miR-375 in HCC treatment. Excellent stability and biocompatibility of AuNPs displayed in this study warrant more extensive investigation of its application in delivery miRNA or siRNA. Our study also highlights the therapeutic potential of miR-375 in HCC treatment and support the development of more effective therapeutic strategies that target miR-375 (or other dysregulated miRNAs) by nanotechnology.

## MATERIALS AND METHODS

### Preparation of AuNPs and AuNP-miR-375

AuNPs were synthesized by using Frence method [20]. MiR-375 mimics and the negative control miR-NC were synthesised by Guangzhou Ribobio Company (Guangzhou, China). For AuNP-miR-375 preparation, thiolated miR-375 was added to 10 nM solution of AuNPs at a ratio of 1 nmol RNA per 500 μL AuNP solution supplemented with 0.1% Tween-20. After incubation at room temperature (RT) for 5 min, the reacted solution was aged with gradual additions of NaCl over 4 h to bring the final NaCl concentration to 0.5 M [21]. Functionalized AuNPs were separated from free RNA strands via centrifugation, supernatant removal, and addition of phosphate buffered saline (PBS) at 4°C, which was repeated twice for further purification. The concentratin of miR-375 in AuNP-miR-375 solution were determined following release of the miRNA from the NPs with dithiothreitol (DTT) using previously described procedures [22]. AuNP and AuNP-miR-375 were characterized by UV-Vis spectrophotometry, dynamic light scattering (DLS) and transmission electron microscope (TEM) according to the manufacturer's instructions.

### Cell treatment and in vitro study

The hepatoma cell lines Hep3B and HepG2 were treated with AuNP-miR-375 (equal as 100 nM miR-375, unless noted otherwise), or equal concentration of AuNP-miR-NC as negative group, or media control, and then harvested for further study 48 h later. Cellular uptake assay, TaqMan qRT-PCR, wound-healing assay, cell migration and invasion, clone formation, cell viability and proliferation, cell apoptosis and Western blot analyses were performed as described before and detailed in Supplementary Materials and Methods [2, 9, 23].

### Animal experiment

FVB/N and BALB/c nude mice were purchased from Beijing HFK Bioscience Co. Ltd. (Beijing, China) and bred under pathogen-free conditions. All animal experiments were carried out in accordance with the Guide for the Care and Use of Laboratory Animals of Tongji Medical College. For xenograft tumor model, subcutaneous tumors were established by inoculation of 5 × 10^6^ HepG2 cells in the front armpit of the BALB/c nude mice (male 5-6 weeks old and 18-20 g). For anti-tumor study in xenograft tumor model, AuNP-miR-375 or AuNP-miR-NC was injected via intratumoral injection when the tumor volume reached about 100 mm^3^, at a dose of 4 nmol/kg miRNA for 4 times at day 7, 10, 14, 17 after HepG2 cells inoculation. Tumor sizes and mouse weights were measured simultaneously. Tumor volume (V) was monitored by measuring the length (L) and width (W) with vernier caliper and calculated with the formula V= (L×W^2^) ×0.5. At the end of the experiment, mice were sacrificed and tumors were collected and photographed. For *in vivo* imaging, the tumor bearing BALB/c nude mice (n = 3) were injected with a single dose of AuNP-miR-375 (equal as 4 nmol/kg miRNA) via tail vein when the tumors reached ∼500 mm^3^. The mice were imaged in a small animal imaging system (Bruker In-vivo FX Pro, CA, USA) by X-ray and fluorescence at 1, 4, 8 h after injection. After that, the mice were sacrificed and organs were collected for *ex vivo* tissue imaging. For toxicology examination of Au-NP-miR-375, FVB/N mice were administrated with AuNP-miR-375 suspensions (equal as 8 nmol/kg miR-375) intravenously via tail vein. The heart, liver, spleen, lung, and kidney of the mice euthanized at 2 or 7 days post injection were collected and then processed for routine histological examination. For primary tumor model, Akt/Ras induced primary HCC mice were generated by hydrodynamic injection of plasmids carrying these two genes into FVB/N mice (6 to 8 weeks old and 18 - 20 g) as described previously [19, 23, 24]. Tumors generally formed in mice 2 weeks after Akt/Ras injection. Then mice were given tail vein injections of AuNP-miR-NC or AuNP-miR-375 (4 nmol/kg miR-375), once every other day for total 3 weeks. Subsequently, the mice were sacrificed and the livers were photographed and examined. Resected tumor tissues from xenograft mice and primary HCC mice were then tested by hematoxylin and eosin (HE), immunohistochemistry and TUNEL staining as described previously by us [2, 25, 26].

### Ethic statement

The study was approved by the local research ethics committee at the Tongji Hospital of Huazhong University of Science and Technology. The methods used in this study were carried out in accordance with the approved guidelines.

### Statistical analysis

All data are expressed as mean ± standard error from 3 separate experiments performed in triplicate except otherwise noted. The differences between groups were analyzed by Student's t test and P < 0.05 was considered to be statistically significant.

## SUPPLEMENTARY DATA



## References

[1] Bruix J, Sherman M (2011). American Association for the Study of Liver D. Management of hepatocellular carcinoma: an update. Hepatology.

[2] He XX, Chang Y, Meng FY, Wang MY, Xie QH, Tang F (2012). MicroRNA-375 targets AEG-1 in hepatocellular carcinoma and suppresses liver cancer cell growth in vitro and in vivo. Oncogene.

[3] Tsukamoto Y, Nakada C, Noguchi T, Tanigawa M, Nguyen LT, Uchida T (2010). MicroRNA-375 is downregulated in gastric carcinomas and regulates cell survival by targeting PDK1 and 14-3-3zeta. Cancer Res.

[4] Mathé EA, Nguyen GH, Bowman ED, Zhao Y, Budhu A, Schetter AJ (2009). MicroRNA expression in squamous cell carcinoma and adenocarcinoma of the esophagus: associations with survival. Clinical Cancer Research.

[5] Zhou J, Song S, Cen J, Zhu D, Li D, Zhang Z (2012). MicroRNA-375 is downregulated in pancreatic cancer and inhibits cell proliferation in vitro. Oncol Res.

[6] Yan JW, Lin JS, He XX (2014). The emerging role of miR-375 in cancer. International journal of cancer Journal international du cancer.

[7] Chang Y, Yan W, He X, Zhang L, Li C, Huang H (2012). miR-375 inhibits autophagy and reduces viability of hepatocellular carcinoma cells under hypoxic conditions. Gastroenterology.

[8] He XX, Kuang SZ, Liao JZ, Xu CR, Chang Y, Wu YL (2015). The regulation of microRNA expression by DNA methylation in hepatocellular carcinoma. Molecular bioSystems.

[9] Liu AM, Poon RT, Luk JM (2010). MicroRNA-375 targets Hippo-signaling effector YAP in liver cancer and inhibits tumor properties. Biochemical and biophysical research communications.

[10] Tao J, Ji J, Li X, Ding N, Wu H, Liu Y, Wang XW, Calvisi DF, Song G, Chen X (2015). Distinct anti-oncogenic effect of various microRNAs in different mouse models of liver cancer. Oncotarget.

[11] Thakor AS, Gambhir SS (2013). Nanooncology: the future of cancer diagnosis and therapy. CA: a cancer journal for clinicians.

[12] Patra CR, Bhattacharya R, Mukhopadhyay D, Mukherjee P (2010). Fabrication of gold nanoparticles for targeted therapy in pancreatic cancer. Adv Drug Deliv Rev.

[13] Hao L, Patel PC, Alhasan AH, Giljohann DA, Mirkin CA (2011). Nucleic acid-gold nanoparticle conjugates as mimics of microRNA. Small.

[14] Rosi NL, Giljohann DA, Thaxton CS, Lytton-Jean AK, Han MS, Mirkin CA (2006). Oligonucleotide-modified gold nanoparticles for intracellular gene regulation. Science.

[15] Seferos DS, Prigodich AE, Giljohann DA, Patel PC, Mirkin CA (2009). Polyvalent DNA nanoparticle conjugates stabilize nucleic acids. Nano Lett.

[16] Massich MD, Giljohann DA, Schmucker AL, Patel PC, Mirkin CA (2010). Cellular response of polyvalent oligonucleotide-gold nanoparticle conjugates. ACS Nano.

[17] Massich MD, Giljohann DA, Seferos DS, Ludlow LE, Horvath CM, Mirkin CA (2009). Regulating immune response using polyvalent nucleic acid-gold nanoparticle conjugates. Mol Pharm.

[18] Chang Y, Li B, Xu X, Shen L, Bai H, Gao F (2016). Lentivirus-Mediated Knockdown of Astrocyte Elevated Gene-1 Inhibits Growth and Induces Apoptosis through MAPK Pathways in Human Retinoblastoma Cells. PloS one.

[19] Ho C, Wang C, Mattu S, Destefanis G, Ladu S, Delogu S (2012). AKT (v-akt murine thymoma viral oncogene homolog 1) and N-Ras (neuroblastoma ras viral oncogene homolog) coactivation in the mouse liver promotes rapid carcinogenesis by way of mTOR (mammalian target of rapamycin complex 1), FOXM1 (forkhead box M1)/SKP2, and c-Myc pathways. Hepatology.

[20] Frens G (1973). Controlled nucleation for the regulation of the particle size in monodisperse gold suspensions. Nature.

[21] Wu XA, Choi CH, Zhang C, Hao L, Mirkin CA (2014). Intracellular fate of spherical nucleic acid nanoparticle conjugates. Journal of the American Chemical Society.

[22] Hurst SJ, Lytton-Jean AK, Mirkin CA (2006). Maximizing DNA loading on a range of gold nanoparticle sizes. Analytical chemistry.

[23] Yang T, Zhao P, Rong Z, Li B, Xue H, You J (2016). Anti-tumor Efficiency of Lipid-coated Cisplatin Nanoparticles Co-loaded with MicroRNA-375. Theranostics.

[24] Chen X, Calvisi DF (2014). Hydrodynamic transfection for generation of novel mouse models for liver cancer research. Am J Pathol.

[25] He XX, Zhang YN, Yan JW, Yan JJ, Wu Q, Song YH (2016). CP-31398 inhibits the growth of p53-mutated liver cancer cells in vitro and in vivo. Tumour Biol.

[26] Yan JJ, Zhang YN, Liao JZ, Ke KP, Chang Y, Li PY, Wang M, Lin JS, He XX (2015). MiR-497 suppresses angiogenesis and metastasis of hepatocellular carcinoma by inhibiting VEGFA and AEG-1. Oncotarget.

