# Supplementary material for: Gold nanoparticles delivered miR-375 for treatment of hepatocellular carcinoma
Source: Oncotarget. 2016 Nov 17;7(52):86675–86. doi: 10.18632/oncotarget.13431 (PMC5349944; doi:10.18632/oncotarget.13431)
Supplement: Supplementary file 1 [file oncotarget-07-86675-s001.pdf]

Gold nanoparticles delivered miR-375 for treatment of hepatocellular carcinoma

SUPPLEMENTARY DATA

Materials

$HAuCl_4$, diethyl pyrocarbonate (DEPC), paraformaldehyde (PFA), propidium iodide (PI), Tween-20, TRIzol, trisodium citrate dihydrate and poly (ethylene glycol) bis (amine) were purchased from Sigma Aldrich (St. Louis, MO, USA). 4',6-diamidino-2-phenylindol (DAPI) was from Biosharp (Hefei, China). Dulbecco's Modified Eagles' Medium (DMEM), fetal bovine serum (FBS), and RIPA lysis buffer were purchased from Thermo (Thermo Fisher, USA). MiR-375 mimics were designed by us as follows: 3' Thiol-(Spacer 6)-CGCAAAACACGCUCCCCGAGCAGCGCCC-5', 5'-GCGUUUUGUUCGUUCGGCUCGCGUGAGG-Cy3-3', the sequences highlighted by red color refer to mature miR-375. MiR-375 mimics were synthesised by Guangzhou Ribobio Company (Guangzhou, China). The negative control miR-NC was provided and synthesised by Guangzhou Ribobio Company. The sequences of miR-375 in mice and humans were the same.

Preparation of AuNPs

AuNPs were synthesized by using Frence method [1]. In a typical procedure, 50 ml of 1 mM $HAuCl_4$ was added into a three-necked flask under vigorous stirring at 130°C. Next, 4 ml of 1% sodium citrate was added to the $HAuCl_4$ solution. The color of the solution changed from faint yellow to wine red within 2 min, indicating the formation of AuNPs. Then the solution continued to be stirred for 15 min. As-formed AuNP solution was resuspended with 0.1% DEPC to remove RNase and then autoclaved at 121°C for 30 min to yield sterile AuNPs.

Characterization of AuNP and AuNP-miR-375

AuNP and AuNP-miR-375 were characterized by UV-Vis spectrophotometry (756 PC, Shanghai Spectrum Instruments, Shanghai, China). Particle size, polydispersity and zeta potential of AuNP and AuNP-miR-375 were measured by DLS (Zeta Plus, Brookhaven Instruments, USA) according to the manufacturer's instructions. All measurements were carried out at room temperature. Each parameter was measured 3 times; average values and standard deviations were calculated. The shapes and diameters of the AuNP and AuNP-miR-375 particles were determined on a TEM (JEOL 100CX II TEM, Japan) at an accelerating voltage of 200 keV.

Cell culture and treatment

The human hepatoma cell lines Hep3B and HepG2 were obtained from the China Center for Type Culture Collection at Wuhan University (Wuhan, China), where the cell lines were authenticated by STR profiling before distribution. The cells were cultured and stored according to supplier's instructions. After resuscitation, they were grown in Dulbecco's modified Eagle medium (DMEM) supplemented with 100 ml/l fetal bovine serum (FBS, Sigma, St. Louis, MO) and 100 units/ml of penicillin–streptomycin (Invitrogen, Carlsbad, CA) and never passaged longer than 6 months and tested routinely by Hoechst DNA staining to ensure no mycoplasma contamination. The cells were treated with AuNP-miR-375 (equal as 100 nM miR-375, unless noted otherwise), or equal concentration of AuNP-miR-NC as nagative group, or nothing as blank control, and then harvested for further study 48 h later.

Cellular uptake assay

To measure the cellular uptake of AuNP-miR-375, HepG2 and Hep3B cells were seeded in 96-well plates at a population of 8×10^3 cells per well and allowed to attach for one day. Then cells were incubated with AuNP-miR-375 (50 nM miR-375). After 1, 3, or 6 h incubation, cells were rinsed with PBS 3 times and fixed in 4% PFA in PBS for 15 min. Then cells were stained with 100 mM DAPI for 3 min. Microscopic examination of cells was performed with an Olympus SZX12 fluorescence microscope equipped with a digital camera and connected to a PC running MagnaFire 2.0 camera software (Optronics, Goleta, CA, USA). Pictures were taken at equal exposure times for each sample. For flow cytometry analysis of cellular uptake, cells were seeded in a 12-well plate at a population of 1×10^5 cells per well and then incubated with AuNP-miR-375 (50 nM miR-375) for various times, followed by 3 times rinse. After that, cells were trypsinized and collected for flow cytometry detection. For each test, 2×10^4 cells were detected and 3 parallel wells were detected for each sample.

TaqMan qRT-PCR

Hep3B and HepG2 cells were treated with AuNP-miR-375 or AuNP-miR-NC and then collected for RNA extraction. The expression of mature miR-375 was

detected by TaqMan qRT-PCR as we have described before [2].

Wound-healing assay

Hep3B cells were seeded at a density of 5×10^5 cells per well in 24-well plates and incubated and allowed to reach 100% confluence overnight. A vertical wound was made with a 200 µL pipette tip through the cell monolayer in a sterile environment. Subsequently, used medium was removed and fresh medium with PBS, AuNP-miR-375 (80 nM miR-375), or Au-miR-NC was added in, respectively. Phase contrast images were taken using a Nikon microscope at 0 and 24 h.

Cell migration and invasion assay

The migration and invasion assay was performed by using Transwell insert chambers (6.5 mm in diameter, 8 µm pore size, Corning, USA). For the migration assay, 5×10^4 Hep3B cells were seeded into the upper chamber in serum-free medium containing PBS, AuNP-miR-375 (miR-375 to 80 nM), or Au-miR-NC. Medium containing 10% serum and the same concentration of drugs as the upper chamber were added to lower chambers as well. After 24 h incubation, non-migrating cells in the upper chambers were removed by using a cotton swab and migratory cells were fixed with 4% PFA for 15 min and then stained with 1% crystal violet solution. Cells that migrated to the underside of the membrane were counted using a Nikon microscope. The invasion activity was assayed as well as the migration assay except that transwell polycarbonate membrane inserts were precoated with a layer of diluted basement membrane Matrigel (ECM gel, Sigma, St. Louis, MO).

Clone formation assay

For the clone formation assay, 2000 cells were plated onto plates and were incubated at 37°C. Once the cells grew to visible colonies, the colonies were washed once with PBS and then fixed with 4% PFA for 15 min. Subsequently, the cells were stained with crystal violet and the number of clones per well was counted.

Cell viability and proliferation assay

Hep3B cells were plated in a 96-well plate with 6,000 cells in each well. After 12 h, Cells were treated with AuNP-miR-375 or controls. Then cell morphology and viabilities were detected under an inverted light microscope and cell proliferation assay were performed by the CCK-8 assay according to the manufacturer's instruction.

Flow cytometry analysis of cell apoptosis and western blot analysis

These assays were detected as we have described before [2]. Cells were harvested for protein extraction 48 h after treatment. Lysates from treated cells were collected using RIPA buffer (Sigma R0278, St Louis, MO, USA). The following antibodies were used: anti-AEG-1 (ProteinTech, USA), anti-ATG7 (CST, USA), anti-YAP1 (CST, USA), anti-Bcl-2 (CST, USA), anti-cleaved-Casepase-3 (CST, USA), and anti-α-tubulin (CST, USA).

REFERENCES

1. Volinia S, Calin GA, Liu CG, Ambs S, Cimmino A, Petrocca F, et al. A microRNA expression signature of human solid tumors defines cancer gene targets. Proc Natl Acad Sci U S A. 2006;103:2257-61.
2. He XX, Chang Y, Meng FY, Wang MY, Xie QH, Tang F, et al. MicroRNA-375 targets AEG-1 in hepatocellular carcinoma and suppresses liver cancer cell growth in vitro and in vivo. Oncogene. 2012;31:3357-69.
